# The Groningen electrocardiographic criteria for left ventricular hypertrophy: a sex-specific analysis

**DOI:** 10.1038/s41598-021-83137-9

**Published:** 2021-03-23

**Authors:** M. Yldau van der Ende, Tom Hendriks, Yordi van de Vegte, Erik Lipsic, Harold Snieder, Pim van der Harst

**Affiliations:** 1 The Department of Cardiology, University Medical Center Groningen, University of Groningen, Hanzeplein 1, 9700 RB Groningen, The Netherlands; 2 The Department of Epidemiology, University Medical Center Groningen, University of Groningen, Groningen, The Netherlands

**Keywords:** Cardiac hypertrophy, Risk factors, Epidemiology

## Abstract

The sensitivity of electrocardiogram (ECG) criteria to detect left ventricular hypertrophy (LVH) is low, especially in women. We determined sex-specific sensitivities of ECG-LVH criteria, and developed new criteria, using cardiovascular magnetic resonance imaging (CMR). Sensitivities of ECG-LVH criteria were determined in participants of the UK Biobank (N = 3632). LVH was defined when left ventricular mass was > 95% confidence interval (CI) according to age and sex. In a training cohort (75%, N = 2724), sex-specific ECG-LVH criteria were developed by investigating all possible sums of QRS-amplitudes in all 12 leads, and selecting the sum with the highest pseudo-R^2^ and area under the curve to detect LVH. Performance was assessed in a validation cohort (25%, N = 908), and association with blood pressure change was investigated in an independent cohort. Sensitivities of ECG-LVH criteria were low, especially in women. Newly developed *Groningen-LVH* criterion for women (Q_V2_ + R_I_ + R_V5_ + R_V6_ + S_V2_ + S_V4_ + S_V5_ + S_V6_) outperformed all ECG-LVH criteria with a sensitivity of 42% (95% CI 35–49%). In men, newly developed criterion ((R_I_ + R_V5_ + S_II_ + S_V2_ + S_V6_) × QRS duration) was equally sensitive as 12-lead sum with a sensitivity of 44% (95% CI 37–51%) and outperformed the other criteria. In an independent cohort, the *Groningen-LVH* criteria were strongest associated with change in systolic blood pressure. Our proposed CMR sex-specific *Groningen-LVH* criteria improve the sensitivity to detect LVH, especially in women. Further validation and its association with clinical outcomes is warranted.

## Introduction

Left ventricular (LV) hypertrophy (LVH) is a marker of the pathophysiologic response of the myocardium to chronic pressure or volume overload and is associated with cardiovascular events^1,2^. The electrocardiogram (ECG) is an inexpensive, widely available but imperfect tool to detect LVH^3^. Numerous ECG criteria have been developed to aid in the detection of LVH^4–7^. The accuracy of the ECG for diagnosing LVH has been described to be lower in women compared to men^8^. However, LVH on the ECG is a stronger risk factor for incident cardiovascular events in women than in men^9^.

In 1949, Sokolow and Lyon developed ECG-LVH criteria based on 147 patients with LVH measured by echocardiography^4^. The average blood pressure of the individuals with LVH in this study was 197/117 mmHg, values that are rarely seen in contemporary clinical practice. Also, no distinction was made between sexes. Later on, sex differences of ECG parameters were precisely described^10,11^. Major novelty of the Cornell criteria^5^, published in 1987, was the stratification for sex and age and increased the sensitivity to detect LVH in women. Most recently developed is the Peguero-Lo Presti criterion with sex-specific thresholds for diagnosing LVH^7^. So far, all ECG-LVH criteria have been developed in cohorts that used echocardiography to measure LV mass. Cardiovascular magnetic resonance imaging (CMR) provides more accurate and reproducible estimates of LV mass^12^.

In this study, we aim to determine the accuracy of existing ECG-LVH criteria in 1670 men and 1962 women of the UK Biobank with available CMR and ECG data. LVH was defined based on reference values of LV mass indexed for body surface area (LVMi), measured by CMR^13^. In addition, we aim to develop the first ECG-LVH criteria, the *Groningen-LVH* criteria, for both men and women based on CMR data.

## Methods

### Study design and population

**Figure 1:**
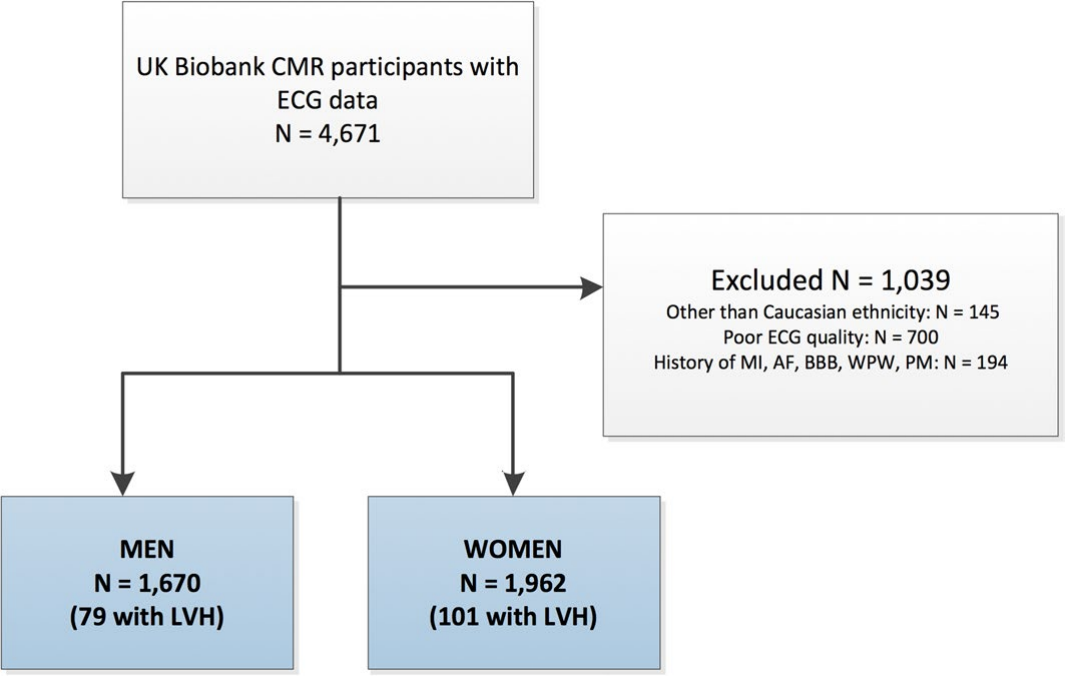
Flowchart of the study population. Al individuals of the UK Biobank CMR substudy with available 12-lead ECG data were included in this study. Individuals with another ethnicity than Caucasian, poor ECG quality or with a history of cardiac disease were excluded from analyses. After exclusion, data of 1670 men and 1962 women was available for analyses. *AF* Atrial fibrillation, *BBB* bundle branch block, *CMR* cardiac magnetic resonance imaging, *ECG* electrocardiogram, *LVH* left ventricular hypertrophy, *PM* pacemaker, *WPW* Wolff-Parkinson-White.

For this study, individuals participating in the CMR substudy of the UK Biobank study with previously determined LV mass on short axis cine series and available 12-lead ECG data were included (N = 4671, Fig. 1)^13^. The study design of the UK Biobank has been described in detail elsewhere^14^. This study was covered by the general ethical approval for UK Biobank studies from the NHS National Research Ethics Service (Ref 11/NW/0382). All methods were performed in accordance with the relevant guidelines and regulations. In brief, the UK Biobank is a population based prospective study established for investigating genetic and non-genetic determinants of diseases. Between 2006 and 2010, 502,664 participants aged between 40–69 years were recruited and signed informed consent. Imaging visits of the UK Biobank were initiated in 2015 in which CMR was performed. In addition, participants underwent a 12-lead resting ECG assessment.

### Cardiovascular magnetic resonance

The UK Biobank’s CMR protocol has been described in detail elsewhere^15^. Long and short axis cine images were acquired on a 1.5 T scanner (MAGNETOM Aera, Syngo Platform VD13A, Siemens Healthcare, Erlangen, Germany). LVH by CMR was defined based on reference values as described by Petersen et al.^13^, using cutoff values of LVMi > 55 g/m^2^ in women, LVMi > 72 g/m^2^ in men aged under 65 years, and LVMi > 70 g/m^2^ in men aged 65 or older^13^.

### Electrocardiography

12-lead ECG data were provided by the UK Biobank in XML format. The ECGs were made using CASE, CardioSoft Version 6 system by a trained research assistant. Amplitudes of the Q wave, R wave and S wave in all 12 leads, as well as QRS duration were predetermined and extracted from the XML files. ECGs automatically evaluated as poor quality (N = 700) were excluded from analyses, as well as ECGs with left or right bundle branch block, atrial fibrillation or flutter, Wolf Parkinson White, or a pacemaker rhythm. Several widely-used ECG-LVH criteria were calculated and examined (Table 1). Additionally, for the Sokolow-Lyon, Cornell, and 12-lead sum criteria, the products with QRS duration were calculated^16^.

**Table 1:** Definitions of established ECG-LVH criteria.

ECG-LVH criteria	Definition	LVH Threshold
Sokolow-Lyon	S_V1_ + R_V5/V6_	> 35 mm
Sokolow-Lyon product	S_V1_ + R_V5/V6_ * QRS duration	Men: > 4,000 mm*ms Women: > 3,000 mm*ms
Cornell	R_aVL_ + S_V3_	Men: > 28 mm Women: > 23 mm
Cornell product	R_aVL_ + S_V3_ * QRS duration	> 2,436 mm*ms
12-lead sum	Sum of the amplitudes of all 12 leads	> 179 mm
12-lead product	Sum of the amplitudes of all 12 leads * QRS duration	> 17,472 mm*ms
Peguero–Lo Presti	Deepest S wave in any single lead + S_V4_	Men: ≥ 28 mm Women: ≥ 23 mm

*mm* millimeter, *ms* millisecond.

### Analytical approach

The first aim of our study was to determine the sex-specific sensitivity and specificity of existing ECG-LVH criteria. Second aim was to develop the first, sex-specific ECG-LVH criteria using CMR data: the *Groningen-LVH* criteria. We randomly divided the study population into a *training* cohort (75%, N = 2724) and a *validation* cohort (25%, N = 908). Third, we aimed to test the performance of the Groningen-LVH criteria in an independent cohort. All statistical analyses were performed using STATA/SE version 15.1 (StataCorp LLC, College Station, Texas, USA). P-values < 0.05 were considered statistically significant.

### Accuracy of established ECG-LVH criteria

Dichotomous baseline characteristics are presented as frequencies and percentages. Continuous variables are summarized by means and standard deviation (SD). Differences between baseline characteristics of men and women with and without LVH were reported; the Chi-square test was used to compare dichotomous variables and differences of continuous variables between groups were evaluated through independent samples t-tests. Sex-specific sensitivity and specificity were reported with related 95% confidence interval (CI). Area under the curve (AUC) analyses, with 95% CI were used to estimate the predicted performance of the existing ECG-LVH criteria.

### Development of sex-specific ECG criteria for diagnosing LVH

Performed steps of the data-driven approach used for the development of our sex-specific ECG-LVH criteria are displayed in Supplementary Fig. 1. First, correlations of LVMi with the Q, R and S wave amplitudes in all 12 leads (36 amplitudes in total) were determined in the *training* population, separately in men and women. Subsequently, for both men and women, all 36 amplitudes were ranked from the amplitude that was highest correlated with LVMi to the amplitude that was lowest correlated. A simple logistic regression analysis was performed with CMR determined LVH as dependent variable and the highest ranked amplitude (the one which was strongest correlated to LVMi) as independent variable, separately for men and women. Next, we added iteratively one extra amplitude, which was next highest ranked, to our model and generated all possible sums of amplitudes (for example, with two amplitudes, three combinations were generated: Amplitude 1, Amplitude 2 and Amplitude 1 + Amplitude 2 etc.). We performed simple logistic regression analyses on LVH with one of the possible sums as independent variable and determined whether one of these models improved the prediction of LVH based on pseudo R^2^ and AUC. Subsequently, the next highest ranked amplitude was added to the model and all possible sums were generated (3 amplitudes give 7 possible combinations, *n* amplitudes give 2^*n*^-1 possible combinations). The final model was selected when subsequently adding two amplitudes to the model did not show an improvement in both sexes based on pseudo R^2^ and AUC.

Finally, all possible generated sums of amplitudes were multiplied with QRS duration to assess whether this further improved the model, based on pseudo R^2^. Threshold of the *Groningen-LVH* criteria were determined using specificities of 90%. AUC analyses were used to estimate the predicted performance of the new ECG-LVH criteria and receiver operating characteristic (ROC) curves were plotted. Statistical significance of the differences in AUC between the proposed criteria and existing criteria was assessed using the Chi-squared and Hanley McNeil tests.

In the *validation* cohort, sensitivity, specificity and AUC were calculated for the proposed ECG-LVH criteria in both men and women and ROC curves were plotted.

### Performance of the developed ECG-LVH criteria in an independent cohort

An increase in systolic blood pressure (SBP) is causally related to an increase in QRS amplitudes^17^. For testing the performance of the *Groningen-LVH* criteria, we examined the association between SBP change (ΔSBP) and QRS amplitudes defined by ECG-LVH criteria for men and women in the independent Lifelines cohort study. The Lifelines cohort study included more than 150.000 individuals of the northern part of the Netherlands^18^. All participants underwent blood pressure measurements and 12-lead ECGs during the baseline and follow-up visit (median follow-up time 3.8 years, interquartile range 3.0–4.6). Linear regression analyses were performed on QRS amplitudes (ECG-LVH criteria during follow-up) with ΔSBP; age, sex and the concordant baseline ECG-LVH criteria as independent variables. Standardized betas of ΔSBP (making both ΔSBP and ECG-LVH criteria unitless) for all models were obtained and compared for all ECG-LVH criteria to determine which ECG-LVH criteria best correlated with a change in blood pressure.

## Results

### Study population

A flow chart for selection of the study population (N = 3632) is shown in Fig. 1. Based on the used cutoff values, 79 men and 101 women with LVH were identified. Supplementary Table 1 shows characteristics of the study population, stratified by the presence of LVH and sex. Values of continuous ECG-LVH criteria as well as LVMi were higher in men compared to women in both the LVH as control group. Among the group with LVH, men and women had comparable age and body mass index and a similar prevalence of hypertension and diabetes.

### Accuracy of ECG-LVH criteria

Several widely-used ECG-LVH criteria were calculated and examined (Table 1). Sensitivity and specificity of ECG-LVH criteria are displayed in Table 2. In men, 12-lead sum had nominally the highest sensitivity (44% (95% CI 37–51%)) followed by the Peguero-Lo Presti criteria (33% (95% CI 26–40%)). In women, the Peguero-Lo Presti criteria showed the highest sensitivity (31% (95% CI 24–38%)), followed by the Cornell criteria (21% (95% CI 15–27%)). Sensitivities of Sokolow-Lyon, Cornell product, 12-lead sum, 12-lead product and Peguero-Lo Presti were nominally lower in women compared to men (Table 2). Sensitivities of the Sokolow-Lyon product and Cornell criteria were higher in women compared to men. Specificity was above 90% for all ECG-LVH criteria and similar in men and women. Accuracy, as measured by the AUC, was nominally highest for the 12-lead product in men (0.77 (95% CI 0.72–0.82)), followed by the 12-lead sum (0.75 (95% CI 0.69–0.81, Supplementary Table 2)). For women, the Peguero-Lo Presti criteria had nominally the highest accuracy with an AUC of 0.75 (95% CI 0.70–0.80), followed by Sokolow-Lyon product (AUC 0.74 (95% CI 0.69–0.79), Supplementary Table 2).

**Table 2:** Sensitivity and specificity of ECG-LVH criteria by sex in complete cohort.

	Sensitivity		Specificity	
	Men	Women	Men	Women
Sokolow-Lyon	32 (25–39)	16 (11–21)	94 (90–97)	99 (98–100)
Sokolow-Lyon product	10 (6–14)	19 (13–25)	99 (98–100)	99 (98–100)
Cornell	6 (3–9)	21 (15–27)	98 (96–100)	96 (93–99)
Cornell product	16 (11–21)	10 (6–14)	96 (93–99)	99 (98–100)
12-lead sum	44 (37–51)	18 (12–24)	92 (88–96)	98 (96–100)
12-lead product	35 (28–42)	9 (5–13)	92 (88–96)	99 (98–100)
Peguero–Lo Presti	33 (26–40)	31 (24–38)	90 (86–94)	93 (89–97)
**Groningen-LVH**	**44 (37–51)**	**42 (35–49)**	**90** (86–94)	**91 (87–95)**

*LVH* left ventricular hypertrophy.

Development of improved ECG criteria for diagnosing LVH.

### Training cohort

**Figure 2:**
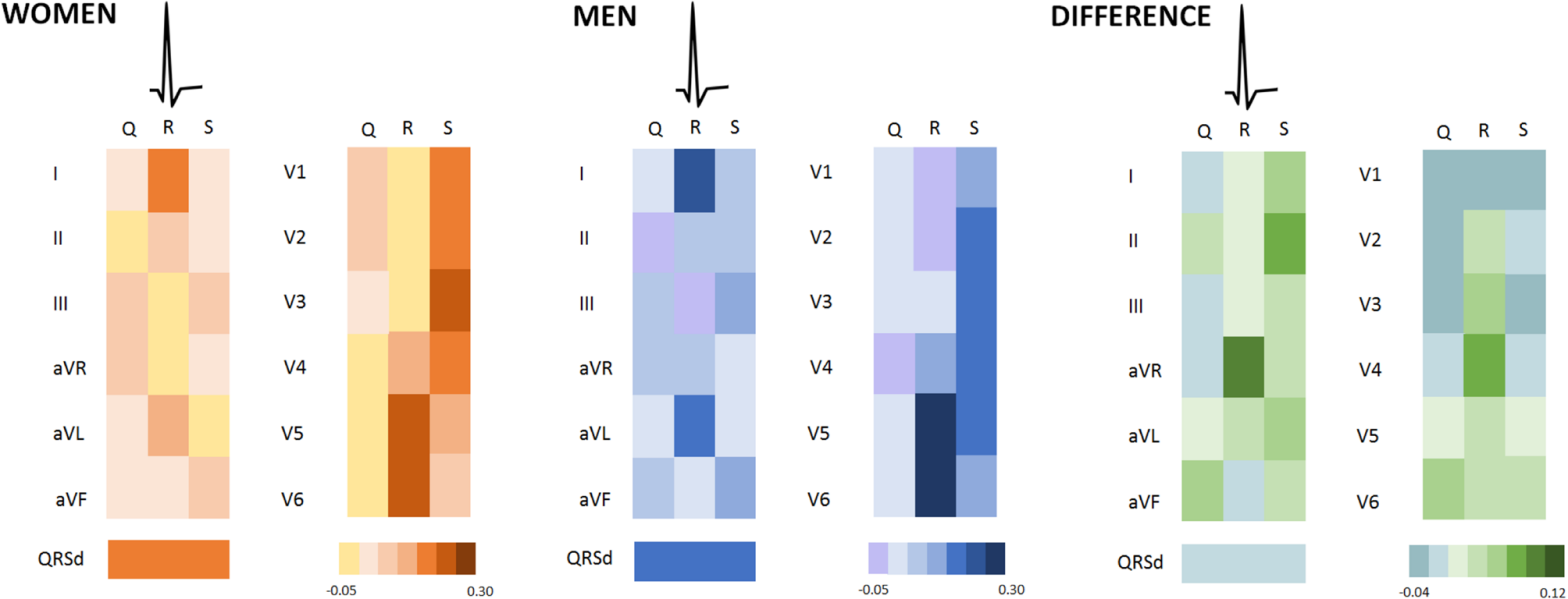
Heatmap of the correlation of Q, R and S waves with LVMi in the training cohort. Correlations are displayed separately for men and women and the difference between men and women. The darker the color in the heatmap, the stronger the correlation of Q, R and S waves with LVMi. Correlation coefficients between − 0.05 (yellow in women, light purple in men) and 0.30 (brown in women, dark blue in men) were observed in women and men. In both men and women, R and S waves are more often highly correlated to LVMi than Q waves. *QRSd* QRS duration.

**Figure 3:**
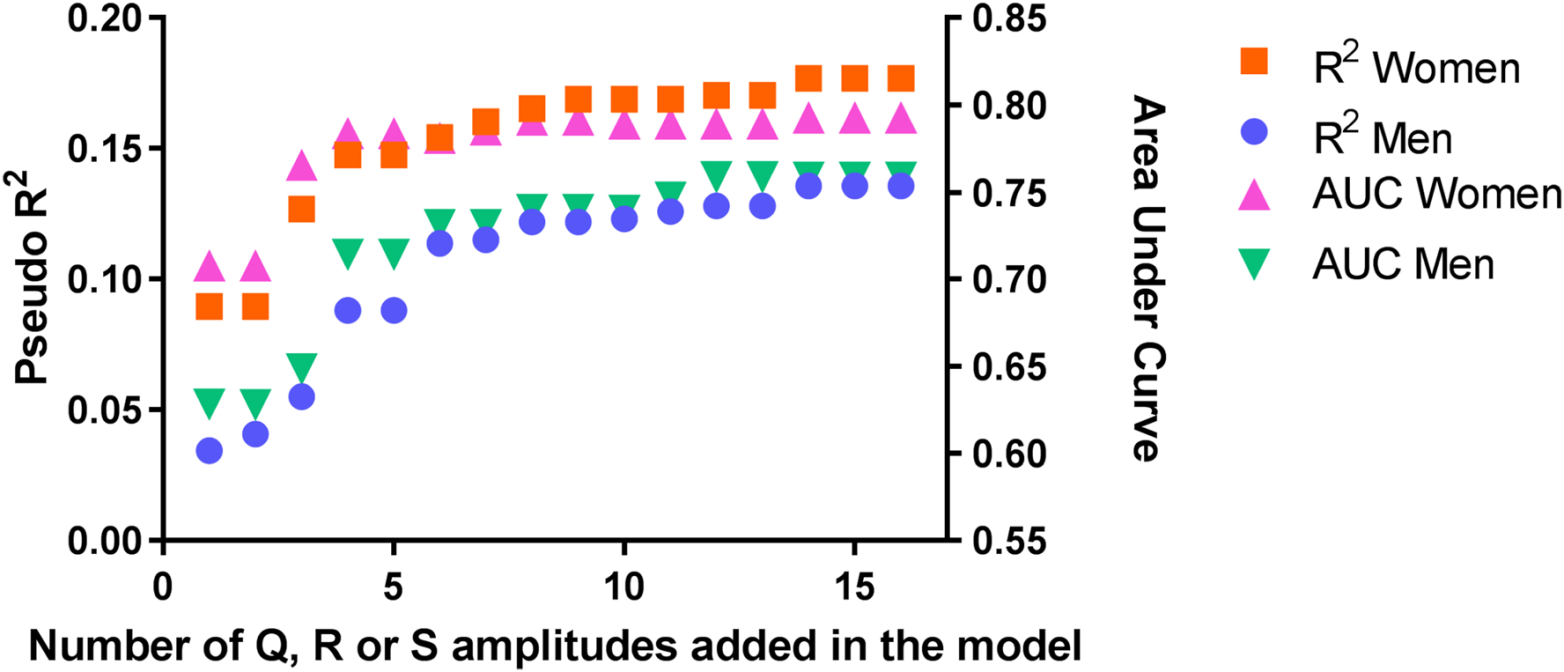
Pseudo R^2^s (orange square for women, blue round for men) and AUCs (pink triangle for women, green triangle for men) of the generated models including all possible sums of amplitudes in logistic regression analyses on LVH. On the X-axis the number of amplitudes added in each model is displayed. On the left Y-axis, the pseudo R^2^s of these models in a logistic regression analyses on CMR determined LVH are reported. The right Y-axis displays the concordant AUCs. *AUC* area under the curve.

In the training cohort (N = 2724), 79 women and 56 men had LVH based on CMR criteria (Supplementary Table 3). In both men and women, R amplitudes in the lateral leads (V4-V6) showed the strongest correlations to LVMi (Fig. 2). Pseudo R^2^ and AUC of a simple logistic regression analysis with the highest correlated amplitudes as independent variables are displayed in Fig. 3. Iteratively adding the next ranked amplitude and generating all possible sums of these amplitudes improved the accuracy of the model based on pseudo R^2^ and AUC (Fig. 3). Adding the 15th and 16th amplitude did not improve the association with LVH anymore in either sex (for women reaching a pseudo R^2^ of 0.177 and AUC of 0.79, for men reaching a pseudo R^2^ of 0.136 and AUC of 0.76, Fig. 3). Supplementary Tables 4 and 5 provide the R^2^ and generated sums of the 16 highest correlated amplitudes. Multiplying the amplitudes with QRS duration improved the prediction in men (pseudo R^2^ of 0.145, AUC 0.78, Supplementary Table 6), but not in women (pseudo R^2^ of 0.168*,* AUC 0.79, Supplementary Table 7). For women, the model with best prediction for LVH was:

$${\rm Q}_{{\rm V}{2}} + {\rm R}_{\rm I} + {\rm R}_{{\rm V}{5}}+ {\rm R}_{{\rm V}{6}} + {\rm S}_{{\rm V}{2}}+ {\rm S}_{{\rm V}{4}}+ {\rm S}_{{\rm V}{5}}+ {\rm S}_{{\rm V}{6}}$$

For men, the following model reached the best prediction:

$${\rm R}_{{\rm I}} + {\rm R}_{\rm V}{5} + {\rm S}_{{\rm II}}+ {\rm S}_{{\rm V}{2}} + {\rm S}_{{\rm V}{6}} \times \text{QRS duration}.$$

**Figure 4:**
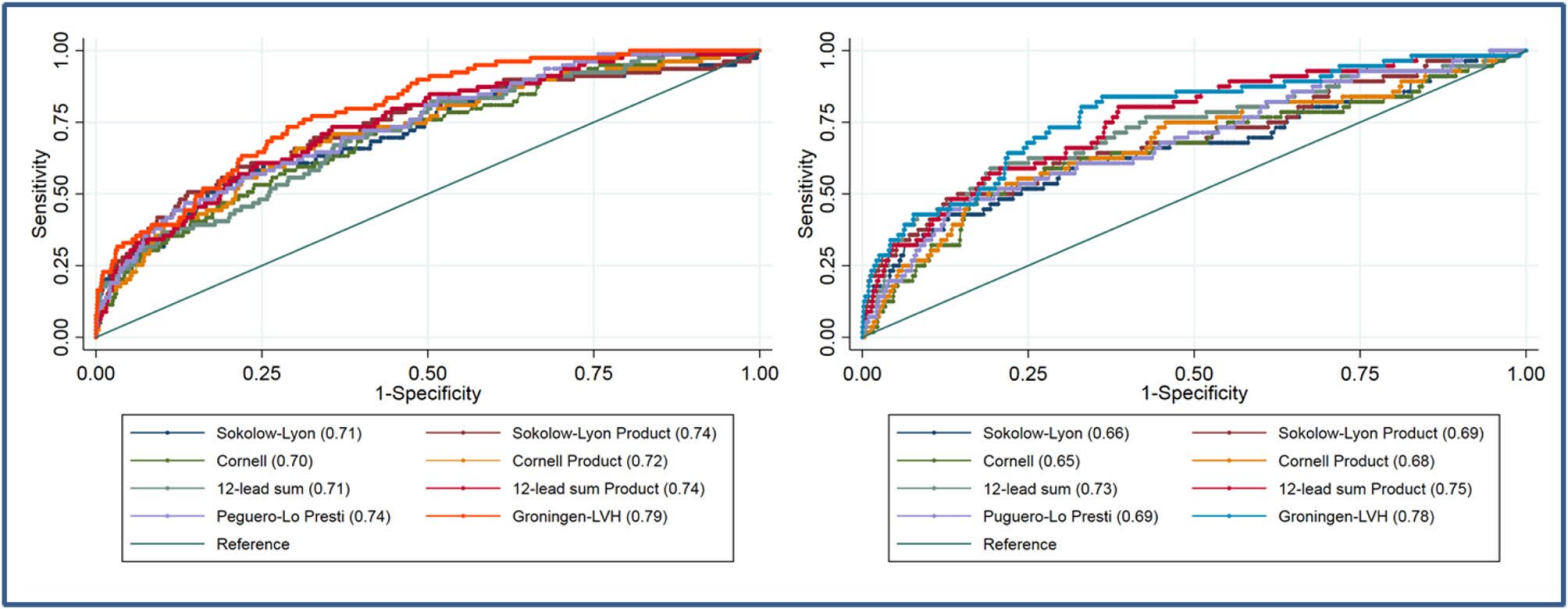
ROC curves for the existing and new developed ECG-LVH criteria in the training cohort. The different colors are ROC curves for each individual ECH-LVH criteria. The left graph shows the ROC curves in women and the right graph the ROC curves in men. The AUC of the *Groningen-LVH* criteria showed the largest AUC for both men and women. In parentheses the AUC is displayed. *LVH* left ventricular hypertrophy.

AUC was 0.79 (0.75–0.84) for *Groningen-LVH* criteria in women, which was nominally the most accurate model compared to existing ECG-LVH criteria (Table 3, Fig. 4). Also, AUC for the *Groningen-LVH* criteria in men was nominally the highest: 0.78 (95% CI 0.71–0.84, Table 3, Fig. 4). Sensitivities of the *Groningen-LVH* criteria were 39% (95% CI 31–47%, with a threshold of 49.5 mm using a specificity of 90%) in women and 43% (95% CI 35–51%, threshold of 4500 mm*milliseconds, specificity of 90%) in men, and were nominally the highest (Table 4). Applying the sex-specific *Groningen-LVH* criteria in the opposite sex, a lower accuracy to detect LVH was identified (pseudo R^2^ of 0.102 and AUC of 0.74 in men; pseudo R^2^ of 0.135 and AUC of 0.77 in women).

**Table 3:** AUCs of the new developed criteria versus AUCs of existing criteria in the training cohort.

	Men AUC	P value	Women AUC	P value
Sokolow-Lyon	0.66 (0.57–0.74)	< 0.001	0.71 (0.65–0.77)	< 0.001
Sokolow-Lyon product	0.69 (0.61–0.77)	< 0.001	0.74 (0.67–0.80)	0.028
Cornell	0.65 (0.57–0.74)	0.018	0.70 (0.64–0.76)	0.003
Cornell product	0.68 (0.60–0.76)	0.026	0.72 (0.66–0.78)	0.170
12-lead sum	0.73 (0.65–0.80)	0.118	0.71 (0.65–0.77)	< 0.001
12-lead product	0.75 (0.69–0.82)	0.139	0.74 (0.68–0.80)	0.180
Peguero-Lo Presti	0.69 (0.61–0.76)	0.051	0.74 (0.68–0.79)	0.012
Groningen-LVH	0.78 (0.71–0.84)		0.79 (0.75–0.84)	

*AUC* area under the curve, *LVH* left ventricular hypertrophy.

**Table 4:** Sensitivity and specificity of the new developed criteria versus sensitivity and specificity of existing criteria in the training cohort.

	Sensitivity		Specificity	
	Men	Women	Men	Women
Sokolow-Lyon	27 (20–34)	15 (9–21)	94 (90–98)	99 (97–100)
Sokolow-Lyon product	11 (6–16)	19 (13–26)	99 (97–100)	99 (97–100)
Cornell	2 (0–4)	22 (15–29)	98 (95–100)	96 (93–99)
Cornell product	14 (8–20)	8 (3–13)	96 (93–99)	99 (97–100)
12-lead sum	41 (33–49)	18 (12–24)	92 (87–97)	99 (97–100)
12-lead product	34 (26–42)	9 (4–14)	91 (86–96)	99 (97–100)
Peguero-Lo Presti	34 (26–42)	32 (24–40)	90 (85–95)	93 (89–97)
**Groningen-LVH**	**43 (35–51)**	**39 (31–47)**	**90 (85–95)**	**90 (85–95)**

*LVH* left ventricular hypertrophy.

### Validation cohort

The validation cohort of 908 individuals, consisted 22 women and 23 men with LVH based on CMR criteria (Supplementary Table 8). Individuals with LVH in the validation cohort were similar to individuals in the training cohort (Supplementary Table 9)*.* Correlation analyses of ECG amplitudes with LVMi showed similar patterns as in the training cohort (Supplementary Fig. 2). For women, the sensitivity of the *Groningen-LVH* criteria was nominally the best (50% (95%CI 35–64%)), followed by the Peguero-Lo Presti criteria (27% (95% CI 14–40%), Table 5). For men, only 12-lead sum had nominally, but not significantly, a higher sensitivity (52% (95% CI 37–67%)), compared to the *Groningen-LVH* criteria with a sensitivity of 48% (95% CI 33–63%), Table 5. Applying the new criteria to the validation cohort, the diagnostic accuracy was nominally higher compared to the accuracy in the training cohort (AUC 0.84 (95% CI 0.76–0.93) in women and 0.82 (95% CI 0.74–0.91%) in men, Table 6 and Supplementary Fig. 3). Combining the training and validation cohort, accuracy of the *Groningen-LVH* criteria for women outperformed all other criteria (Supplementary Table 2), using the Chi-squared test. Using the Hanley McNeil test, accuracy of the *Groningen-LVH* criteria for women was similar to Peguero-Lo Presti criterium. For men, the *Groningen-LVH* criteria had nominally the same sensitivity as 12-lead sum (Table 2). AUC was nominally the largest for the *Groningen-LVH* criteria in men but did not differ significantly from 12-lead sum and 12-lead product (Supplementary Table 2, using Chi-squared test). Using Hanley McNeil test, the *Groningen-LVH* criteria for men did not differ significantly from 12-lead sum, 12-lead product and Sokolow-Lyon product.

**Table 5:** Sensitivity and specificity of the new developed criteria versus sensitivity and specificity of existing criteria in the validation cohort.

	Sensitivity		Specificity	
	Men	Women	Men	Women
Sokolow-Lyon	43 (29–57)	18 (7–30)	94 (87–100)	98 (94–100)
Sokolow-Lyon product	9 (0–17)	18 (7–30)	99 (96–100)	99 (96–100)
Cornell	17 (6–28)	18 (7–30)	99 (96–100)	96 (90–100)
Cornell product	22 (10–34)	18 (7–30)	97 (92–100)	99 (96–100)
12-lead sum	52 (37–67)	18 (7–30)	90 (81–99)	97 (92–100)
12-lead product	39 (25–53)	9 (0–17)	92 (84–100)	99 (96–100)
Peguero-Lo Presti	30 (17–43)	27 (14–40)	91 (83–99)	93 (86–100)
**Groningen-LVH**	**48 (33–63)**	**50 (35–64)**	**91 (83–99)**	**93 (86–100)**

*LVH* left ventricular hypertrophy.

**Table 6:** Area under the curve of the new developed criteria versus AUCs of existing criteria in the validation cohort.

	Men AUC	P value	Women AUC	P value
Sokolow-Lyon	0.81 (0.71–0.91)	0.740	0.72 (0.61–0.82)	0.024
Sokolow-Lyon product	0.82 (0.73–0.91)	0.952	0.77 (0.67–0.87)	0.169
Cornell	0.67 (0.53–0.81)	0.023	0.72 (0.62–0.83)	0.013
Cornell product	0.66 (0.53–0.80)	0.012	0.75 (0.65–0.85)	0.047
12-lead sum	0.82 (0.74–0.91)	0.994	0.72 (0.61–0.83)	< 0.001
12-lead product	0.81 (0.72–0.89)	0.724	0.76 (0.66–0.85)	0.009
Peguero-Lo Presti	0.69 (0.56–0.81)	0.030	0.80 (0.73–0.88)	0.380
**Groningen-LVH**	0.82 (0.74–0.91)		0.84 (0.76–0.93)	

*AUC* area under the curve, *LVH* left ventricular hypertrophy.

### Performance of the new ECG-LVH criteria in an independent cohort

Standardized betas of ΔSBP on ECG-LVH criteria assessed by linear regression analyses are displayed in Supplementary Table 10. An increase of one SD in ΔSBP increases the *Groningen-LVH* criteria with 0.095 SDs in women and with 0.068 SDs in men, which were nominally the largest effects of ΔSBP as compared to other ECG-LVH criteria.

## Discussion

In this study, accuracies of existing ECG-LVH criteria were determined in 1,670 men and 1,962 women participating in the UK Biobank with available CMR-derived LVM measurements and 12-lead ECG data. Sensitivity of established ECG-LVH criteria is low, especially in women. The lower sensitivity of ECG-LVH criteria in women has been reported earlier^19^. Antihypertensive treatment can decrease LVH and improve left ventricular dysfunction^20^. The higher chance of false negative findings of LVH in women may therefore lead to undertreatment of LVH in women and the incidence of preventable cardiovascular events^21^. In the current study, we therefore developed the sex-specific *Groningen-LVH criteria*, which performed significantly better than the previously established criteria in women.

Women have lower ECG signal amplitudes than men^10^, which may be one of the explanations of the lower sensitivity of ECG-LVH criteria in women. Intuitively, the relatively lower QRS voltages in women could be due to the presence of breast tissue. However, it has been reported that breast tissue accounts for less that 1% of the total variation of QRS voltages^22^; a variation that may not be different from the normal day to day variation of ECG voltages^23^. Women with LVH in our study did not have a higher BMI compared to men, another factor that has been described to be inversely associated with sensitivity^19^. In addition to female sex, one study has reported age, blood pressure, relative wall thickness and the use of antihypertensive medication as predictors of this discrepancy^24^. In our study population, there was no difference in age or the presence of hypertension between men and women with LVH. Also, there was no sex interaction for mass to volume ratio between individuals with and without LVH in our study (sex interaction *P*-value 0.32 in the training cohort). Our findings therefore suggest that the sex differences in sensitivity may largely be explained by the lower absolute LVMi in women compared to men and the absence of sex-specific cut off points for most established ECG-LVH criteria.

Most of the ECG-LVH criteria were developed between 1940 and 1990. Since then, major changes have occurred in lifestyle, prevention and treatment of cardiovascular disease. Study populations in whom these criteria are developed may therefore differ from the contemporary population. Furthermore, the established ECG-LVH criteria were developed and validated using echocardiography as reference^4–7^. CMR provides more accurate, precise and reproducible estimates of LV mass^12^. Our reported sensitivities and specificities may therefore be a more precise measure of the performance of ECG criteria in the current general population.

For most of the existing ECG-LVH criteria, no distinction has been made between men and women. Sex differences of ECG amplitudes and durations are nowadays precisely described^10,11^ and suggest different cut off points and/or other criteria for men and women to detect LVH using ECG. Thus, the most important finding of the present study is the development of sex-specific ECG-LVH criteria, of which the accuracy is similar in men and women.

The development of our new sex-specific ECG criteria using CMR data, started with determining the correlation of Q, R and S amplitudes of all 12 leads with LVMi for both men and women. In both sexes, Q waves were less often highly correlated to LVMi than R or S waves. The Q wave is a reflection of the depolarization of the septum, conduction system and endomyocardial fibers of the left ventricle. The R and S wave are related to the depolarization of the myocardial and epicardial wall of the left ventricle^25^. Changes in voltages due to LVH may therefore be better represented in these waves. For both men and women, the most accurate combination of amplitudes was generated with a large proportion of amplitudes of the lateral leads (I, V5 and V6), which represent the electrical activity from the vantage point of the lateral wall of left ventricle. Since the electrical vector of the left ventricle is enhanced in LVH, R-waves in lateral leads and S waves in right sided chest leads (V1, V2) will increase as a result as well. As described by Peguero et al*.* it is plausible that changes in voltage that occur in patients with mild to moderate LVH are better represented by the latter part of the QRS complex, which corresponds to the S wave^7^. Our proposed criteria suggest that both R and S waves are important predictors of LVH and that the sum of a combination of R and S waves is most accurate.

Multiplying the sum of amplitudes with QRS duration improved the accuracy of the model for men, but not the model for women. Molloy et al*.* described the improvement of detecting LVH by the product of QRS duration with voltage^16^. However, the majority of the individuals in the study of Molloy et al. were men and no subgroup analyses was performed to see whether the product of QRS duration improved the prediction in both sexes. In a population with LVH due to underlying valvular regurgitation (*volume* overload which mainly leads to eccentric hypertrophy), accuracy of ECG-LVH criteria improved by multiplying the sum of amplitudes with QRS duration in both men and women^26^. In our study population, the majority (60%) of the individuals with LVH had hypertension and therefore *pressure* overload of the heart. It has been reported that male and female hearts under *pressure* overload respond primarily with concentric hypertrophy. However, women stay more in concentric hypertrophy, whereas men more often develop eccentric hypertrophy^27^. This sex difference in remodeling may be an explanation of the improvement of the ECG-LVH criteria multiplied by QRS duration in men, but not in women. Future studies, investigating ECG-LVH criteria separately for eccentric and concentric hypertrophy, are needed to validate this hypothesis.

## Limitations

For the development of our sex-specific Groningen-LVH criteria we generated the most predictive models instead of the simplest. We are aware that calculation of our developed criteria by hand may be more time consuming than calculating the existing, simple criteria. However, for computer based algorithms used by ECG software, this complexity does not matter and will therefore give the best risk prediction for LVH.

Our proposed LVH criteria for women improved the accuracy for diagnosing LVH in both the *training* and validation cohort. For men, our proposed criteria had nominally the highest sensitivity in the *training* cohort, but 12-leadsum had nominally the highest sensitivity in the *validation* cohort. In the complete cohort, the sensitivity and AUC of our proposed criteria in men was as high as the 12-lead sum. Further validation in a larger population is therefore warranted for our proposed male criterion.

As we excluded individuals with a non-Caucasian ethnicity, and individuals with previous cardiovascular disease, the accuracy of our proposed criteria might not be generalizable and needs to be validated in these populations.

## Conclusion

The sensitivity of existing ECG criteria to detect LVH measured by CMR is low, especially in women. Our proposed sex-specific *Groningen-LVH* criteria are the first criteria generated with CMR as reference, and improve the accuracy to detect LVH, especially in women. Further validation of our criteria in independent cohorts is warranted.

## Supplementary Information

Supplementary Information 1.

Supplementary Information 2.

## Data Availability

The data that support the findings of this study are available from UK Biobank, but restrictions apply to the availability of these data, which were used under license for the current study, and so are not publicly available. Data are however available from the authors upon reasonable request and with permission of the UK Biobank.

## Abbreviations

AUC: Area under the curve
ECG: Electrocardiogram
CI: Confidence interval
CMR: Cardiovascular magnetic resonance
LV: Left ventricle
LVH: Left ventricular hypertrophy
LVMi: Left ventricular mass indexed to body surface area
ROC: Receiver operating characteristic
SBP: Systolic blood pressure
SD: Standard deviation
