# Supplementary material for: The Groningen electrocardiographic criteria for left ventricular hypertrophy: a sex-specific analysis
Source: Sci Rep. 2021 Mar 23;11:6662. doi: 10.1038/s41598-021-83137-9 (PMC7988153; doi:10.1038/s41598-021-83137-9)

Online Appendix

The Groningen Electrocardiographic Criteria for Left Ventricular Hypertrophy: a Sex-Specific Analysis

M. Yldau van der Ende, BSc[a], Tom Hendriks, M.D.[a], Yordi van de Vegte, BSc[a] , Erik Lipsic, M.D., Ph.D.[a], Harold Snieder, Ph.D.[b], Pim van der Harst, M.D., Ph.D.[a]

Online Table 1. Characteristics of the study population in the complete population of the UK Biobank imaging substudy.

	LVH<br>Men<br>N = 79	Women<br>N = 101	P value	No LVH<br>Men<br>N = 1591	Women<br>N = 1861	P value
Age (years)	61.4 (7.8)	63.0 (7.7)	0.206	62.8 (7.5)	61.8 (7.4)	<0.001
Anthropometry						
Height (cm)	162.4 (6.3)	175.2 (6.9)	<0.001	162.7 (6.4)	176.2 (6.4)	<0.001
BMI (kg/m^2)	27.4 (3.7)	26.4 (5.3)	0.141	27.1 (3.8)	26.3 (4.6)	<0.001
Body surface area (m^2)	2.0 (0.2)	1.7 (0.2)	<0.001	2.0 (0.2)	1.7 (0.2)	<0.001
Risk factors						
Hypertension (%, *n*)	58.2 (46)	54.5 (55)	0.613	38.5 (612)	27.8 (517)	<0.001
Diabetes (%, *n*)	8.9 (7)	7.9 (8)	0.812	5.8 (92)	4.1 (77)	0.021
Hypercholesterolemia (%, *n*)	39.2 (31)	20.8 (21)	0.007	33.4 (531)	19.2 (357)	<0.001
Smoking (current or former) (%, *n*)	55.7 (44)	57.4 (58)	0.816	58.0 (1,591)	49.4 (919)	<0.001
ECG LVH criteria						
Sokolow-Lyon (mm)	30.1 (10.2)	25.6 (8.6)	0.002	23.3 (7.1)	19.9 (6.0)	<0.001
Sokolow-Lyon product (mm*ms)	2888 (1064)	2250 (805)	<0.001	2122 (709)	1641 (563)	<0.001
Cornell (mm)	17.7 (7.2)	15.7 (6.8)	0.060	14.1 (5.5)	11.1 (4.9)	<0.001
Cornell product (mm*ms)	1705 (748)	1481 (721)	0.044	1300 (578)	998 (470)	<0.001
12-lead sum (mm)	170 (37)	145 (36)	<0.001	140 (28)	120 (25)	<0.001
12-lead product (mm*ms)	16390 (4390)	12813 (3905)	<0.001	12755 (3335)	9929 (2717)	<0.001
Peguero-Lo Presti (mm)	24.7 (9.7)	21.3 (7.8)	0.010	18.9 (7.0)	15.3 (5.0)	<0.001
QRS duration (ms)	96.4 (14.9)	88.2 (12.5)	<0.001	91.0 (12.8)	82.6 (11.9)	<0.001
LVMi (g/m^2)	77.3 (6.9)	59.7 (5.6)	<0.001	52.1 (7.8)	41.7 (5.9)	<0.001
LVEDVi (mL/m^2)	98.3 (16.8)	84.7 (16.8)	<0.001	81.0 (13.8)	71.5 (10.8)	<0.001
Mass to Volume Ratio (g/mL)	0.81 (0.15)	0.73 (0.14)	<0.001	0.66 (0.12)	0.59 (0.10)	<0.001

cm = centimeter, g = gram, LVEDVi = left ventricular end diastolic volume indexed for body surface area, LVMi = left ventricular mass indexed for body surface area, mL = milliliter, mm = millimeter, ms = millisecond

Online Table 2. Area under curves for existing ECG LVH criteria in men and women.

AUC **complete** cohort

	**MEN**<br>**AUC**	**P value**	**WOMEN**<br>**AUC**	**P value**
Sokolow-Lyon	0.70 (0.63 – 0.77)	0.002	0.71 (0.66 – 0.77)	<0.001
Sokolow-Lyon product	0.73 (0.66 – 0.79)	0.004	0.74 (0.69 – 0.79)	0.007
Cornell	0.66 (0.59 – 0.73)	0.002	0.71 (0.65 – 0.76)	<0.001
Cornell product	0.67 (0.60 – 0.74)	0.002	0.72 (0.67 – 0.78)	0.002
12-lead sum	0.75 (0.69 – 0.81)	0.174	0.72 (0.66 – 0.76)	<0.001
12-lead product	0.77 (0.72 – 0.82)	0.212	0.71 (0.66 – 0.76)	<0.001
Peguero-Lo Presti	0.69 (0.62 – 0.75)	0.007	0.75 (0.70 – 0.80)	0.006
**Groningen-LVH**	0.79 (0.74 – 0.84)		0.81 (0.76 – 0.85)	

AUC = area under the curve, LVH = left ventricular hypertrophy

Online Table 3. Characteristics of the **training** population

	LVH<br>Men<br>N = 56	Women<br>N = 79	P value	No LVH<br>Men<br>N = 1182	Women<br>N = 1407	P value
Age (years)	61.5 (7.9)	62.6 (7.5)	0.430	62.8 (7.5)	61.7 (7.5)	<0.001
Anthropometry						
Height (cm)	175.0 (6.3)	162.7 (6.4)	<0.001	176.2 (6.4)	162.9 (6.5)	<0.001
BMI (kg/m^2)	27.6 (3.5)	26.7 (5.6)	0.272	27.1 (3.7)	26.1 (4.5)	<0.001
Body surface area (m^2)	2.00 (0.16)	1.75 (0.18)	<0.001	2.00 (0.16)	1.74 (0.15)	<0.001
Risk factors						
Hypertension (%, *n*)	64.3 (36)	57.0 (45)	0.392	39.4 (465)	27.9 (392)	<0.001
Diabetes (%, *n*)	8.9 (5)	8.9 (7)	0.989	6.0 (71)	3.9 (55)	0.0013
Hypercholesterolemia (%, *n*)	42.9 (24)	20.3 (16)	0.005	33.2 (392)	19.1 (268)	<0.001
Smoking (current or former) (%, *n*)	58.9 (33)	55.7 (44)	0.709	49.3 (694)	58.0 (685)	<0.001
ECG LVH criteria						
Sokolow-Lyon (mm)	29.2 (10.7)	25.6 (8.3)	0.028	23.4 (7.0)	20.0 (6.0)	<0.001
Sokolow-Lyon product (mm*ms)	2846 (1170)	2250 (819)	<0.001	2131 (716)	1656 (556)	<0.001
Cornell (mm)	17.4 (6.7)	15.6 (6.6)	0.112	4.1 (5.6)	11.1 (4.9)	<0.001
Cornell product (mm*ms)	1695 (719)	1464 (720)	0.069	1297 (588)	1000 (472)	<0.001
12-lead sum (mm)	168 (38.4)	146 (36.4)	0.001	139 (28.0)	120 (24.8)	<0.001
12-lead product (mm*ms)	16321 (4828)	12801 (3960)	<0.001	12753 (3395)	9981 (2701)	<0.001
Peguero-Lo Presti (mm)	24.6 (9.5)	21.2 (7.9)	0.024	18.9 (7.1)	15.3 (5.0)	<0.001
QRS duration (ms)	97.6 (16.8)	88.0 (13.5)	<0.001	90.8 (13.6)	92.7 (12.0)	<0.001
LVMi (g/m^2)	78.1 (7.3)	59.6 (5.8)	<0.001	52.0 (7.9)	41.7 (6.0)	<0.001
LVEDVi (mL/m^2)	98.8 (16.0)	85.0 (17.7)	<0.001	80.7 (13.7)	71.8 (10.8)	<0.001
Mass to Volume Ratio (g/mL)	0.81 (0.14)	0.73 (0.14)	0.001	0.66 (0.12)	0.59 (0.10)	<0.001

cm = centimeter, LVEDVi = left ventricular end diastolic volume indexed for body surface area, LVMi = left ventricular mass indexed for body surface area, mL = milliliter, mm = millimeter, ms = millisecond

Online Tables 4-7: Excel files

Online Table 8. Characteristics of the **validation** population

	LVH<br>Men<br>N = 23	Women<br>N = 22	P value	No LVH<br>Men<br>N = 409	Women<br>N = 454	P value
Age (years)	64.1 (8.3)	61.2 (7.8)	0.244	63.0 (7.4)	62.2 (7.2)	0.115
Anthropometry						
Height (cm)	176.0 (8.3)	161.3 (6.2)	<0.001	176.4 (6.3)	162.0 (6.0)	<0.001
BMI (kg/m^2)	27.0 (4.4)	24.4 (3.9)	0.202	27.3 (4.0)	26.7 (5.0)	0.055
Body surface area (m^2)	2.00 (0.24)	1.70 (0.14)	<0.001	2.01 (0.16)	1.74 (0.15)	<0.001
Risk factors						
Hypertension (%, *n*)	43.5 (10)	45.5 (10)	0.894	35.9 (147)	24.5 (125)	0.008
Diabetes (%, *n*)	8.7 (2)	4.6 (1)	0.577	5.1 (32)	4.9 (22)	0.846
Hypercholesterolemia (%, *n*)	30.4 (7)	22.7 (5)	0.559	34.0 (139)	19.6 (89)	<0.001
Smoking (current or former) (%, *n*)	47.8 (11)	63.6 (12)	0.286	58.0 (237)	49.6 (225)	0.014
ECG LVH criteria						
Sokolow-Lyon (mm)	32.2 (8.7)	25.6 (9.6)	0.019	22.9 (7.1)	19.3 (6.2)	<0.001
Sokolow-Lyon product (mm*ms)	2992 (758)	2252.8 (770)	0.002	2096 (692)	1596 (580)	<0.001
Cornell (mm)	16.4 (7.5)	18.4 (8.5)	0.391	14.2 (5.4)	11.2 (5.0)	<0.001
Cornell product (mm*ms)	1729 (833)	1540 (737)	0.424	1310 (548)	994 (461)	<0.001
12-lead sum (mm)	177.5 (32.1)	144.2 (36.8)	0.002	139.0 (28.5)	117.9 (26.1)	<0.001
12-lead product (mm*ms)	16561 (3163)	12857 (3793)	<0.001	12764 (3159)	9766 (2762)	<0.001
Peguero-Lo Presti (mm)	24.9 (10.6)	21.8 (7.4)	0.253	18.9 (6.9)	15.2 (4.9)	<0.001
QRS duration (ms)	93.5 (8.7)	88.8 (8.2)	0.072	91.5 (10.3)	82.3 (11.5)	<0.001
LVMi (g/m²)	75.3 (5.5)	60.0 (5.3)	<0.001	52.6 (7.8)	41.8 (5.8)	<0.001
LVEDVi (mL/m²)	97.1 (18.9)	83.7 (13.6)	<0.001	81.8 (14.2)	70.8 (10.8)	<0.001
Mass to Volume Ratio (g/mL)	0.80 (0.17)	0.74 (0.14)	0.138	0.66 (0.12)	0.60 (0.10)	<0.001

cm = centimeter, LVEDVi = left ventricular end diastolic volume indexed for body surface area, LVMi = left ventricular mass indexed for body surface area, mL = milliliter, mm = millimeter, ms = millisecond

Online Table 9. Characteristics of individuals with and without left ventricular hypertrophy in the **training** and **validation** cohort.

	**LVH**<br>Training<br>N = 135	Validation<br>N =45	P value	**No LVH**<br>Training<br>N = 2589	Validation<br>N = 863	P value
Age (years)	62.2 (7.7)	62.6 (8.1)	0.766	62.2 (7.5)	62.6 (7.3)	0.248
Anthropometry						
Height (cm)	167.8 (8.7)	168.8 (10.4)	0.541	169.0 (9.2)	168.8 (9.4)	0.645
BMI (kg/m^2)	27.0 (4.8)	26.3 (4.2)	0.320	26.6 (4.2)	27.0 (4.5)	**0.016**
Body surface area (m^2)	1.85 (0.21)	1.85 (0.25)	0.947	1.86 (0.20)	1.87 (0.21)	0.330
Risk factors						
Hypertension (%, *n*)	60.0 (81)	44.4 (20)	0.069	33.1 (857)	31.5 (272)	0.390
Diabetes (%, *n*)	8.9 (12)	6.7 (3)	0.640	4.9 (126)	5.0 (43)	**0.019**
Hypercholesterolemia (%, *n*)	29.6 (40)	26.7 (12)	0.704	25.5 (660)	26.4 (228)	0.590
Smoking (current or former) (%, *n*)	57.0 (77)	55.6 (25)	0.862	53.3 (1379)	53.5 (462)	0.890
ECG LVH criteria						
Sokolow-Lyon (mm)	27.1 (9.5)	29.0 (9.6)	0.243	21.6 (6.7)	21.0 (6.9)	**0.036**
Sokolow-Lyon product (mm*ms)	2497 (1019)	2630 (842)	0.429	1873 (676)	1833 (683)	0.134
Cornell (mm)	16.3 (6.7)	17.4 (8.0)	0.357	12.5 (5.4)	12.6 (5.4)	0.649
Cornell product (mm*ms)	1560 (726)	1637 (117)	0.550	1135 (548)	1144 (528)	0.697
12-lead sum (mm)	154.7 (38.7)	161.2 (38.0)	0.328	129.2 (28.0)	127.9 (29.2)	0.256
12-lead product (mm*ms)	14261 (4661)	14750 (3821)	0.528	11246 (3336)	11187 (3313)	0.648
Peguero-Lo Presti (mm)	22.6 (8.8)	23.4 (9.2)	0.599	17.0 (6.3)	17.0 (6.2)	0.983
QRS duration (ms)	92.0 (15.6)	91.2 (8.7)	0.753	86.4 (13.3)	86.7 (11.8)	0.518
LVMi (g/m^2)	67.3 (11.2)	67.8 (1.4)	0.777	46.4 (8.6)	46.9 (8.7)	0.131
LVEDVi (mL/m^2)	90.7 (18.3)	90.5 (17.7)	0.948	75.9 (13.0)	76.0 (13.7)	0.785
Mass to Volume Ratio (g/mL)	0.76 (0.15)	0.77 (0.15)	0.687	0.62 (0.11)	0.63 (0.11)	0.147

cm = centimeter, LVEDVi = left ventricular end diastolic volume indexed for body surface area, LVMi = left ventricular mass indexed for body surface area, mL = milliliter, mm = millimeter, ms = millisecond

Online Table 10. Linear regression analyses on QRS amplitudes (ECG-LVH criteria during follow-up) with ΔSBP, age, sex and the concordant baseline ECG-LVH criteria as independent variables in the independent Lifelines cohort.

	**Standardized beta Δ SBP MEN**	**Standardized beta Δ SBP WOMEN**
Sokolow-Lyon	0.048	0.066
Sokolow-Lyon product	0.049	0.062
Cornell	0.063	0.064
Cornell product	0.060	0.024
12-lead sum	0.051	0.063
12-lead product	0.050	0.055
Peguero-Lo Presti	0.062	0.070
**Groningen-LVH**	**0.068**	**0.095**

Standardized betas display the SD change of the ECG-LVH criteria that could be explained by a SD difference in systolic blood pressure. SBP = systolic blood pressure, LVH=left ventricular hypertrophy

Online Figure 1. Performed steps of the data-driven approach used for the development of our sex-specific ECG-LVH criteria

1. Determining correlations of 36 **Q,R or S amplitudes** of all 12 lead with **LVMi**

2. **Ranking** of amplitudes based on correlation

   1. highest correlated amplitude
   2. Second highest correlated amplitude
   ~
   35. Second lowest correlated amplitude
   36. Lowest correlated amplitude

3. Logistic regression analyses on **LVH** with **ranked 1 amplitude** as independent variable

4. Logistic regression analyses on **LVH** with a possible sum of **ranked 1** & **ranked 2** amplitudes as independent variable (3 simple logistic regression analyses)
   1. Ranked 1
   2. **Ranked 2**
   3. Ranked 1 + **2**

5. Logistic regression analyses on LVH with a possible sum of **ranked 1** & **ranked 2** & **ranked 3** amplitudes as independent variable (7 simple logistic regression analyses)
   1. **Ranked 1**
   2. **Ranked 2**
   3. **Ranked 3**
   4. Ranked **1** + **2**
   5. Ranked **1** + **3**
   6. Ranked **2** + **3**
   7. Ranked **1** + **2** + **3**

6. Iteratively adding the next highest ranked amplitude and generating all possible sums for simple logistic regression analyses. The **final model** was chosen when adding two amplitudes did not show improvement based on R^2 and AUC

7. **Multiplying** the final sum with best prediction of LVH with **QRS duration**, to determine whether this further improved the model based on pseudo R^2

Online Figure 2. Heatmap of the correlation of Q, R and S waves with left ventricular mass indexed by body surface area in men and women of the validation cohort.

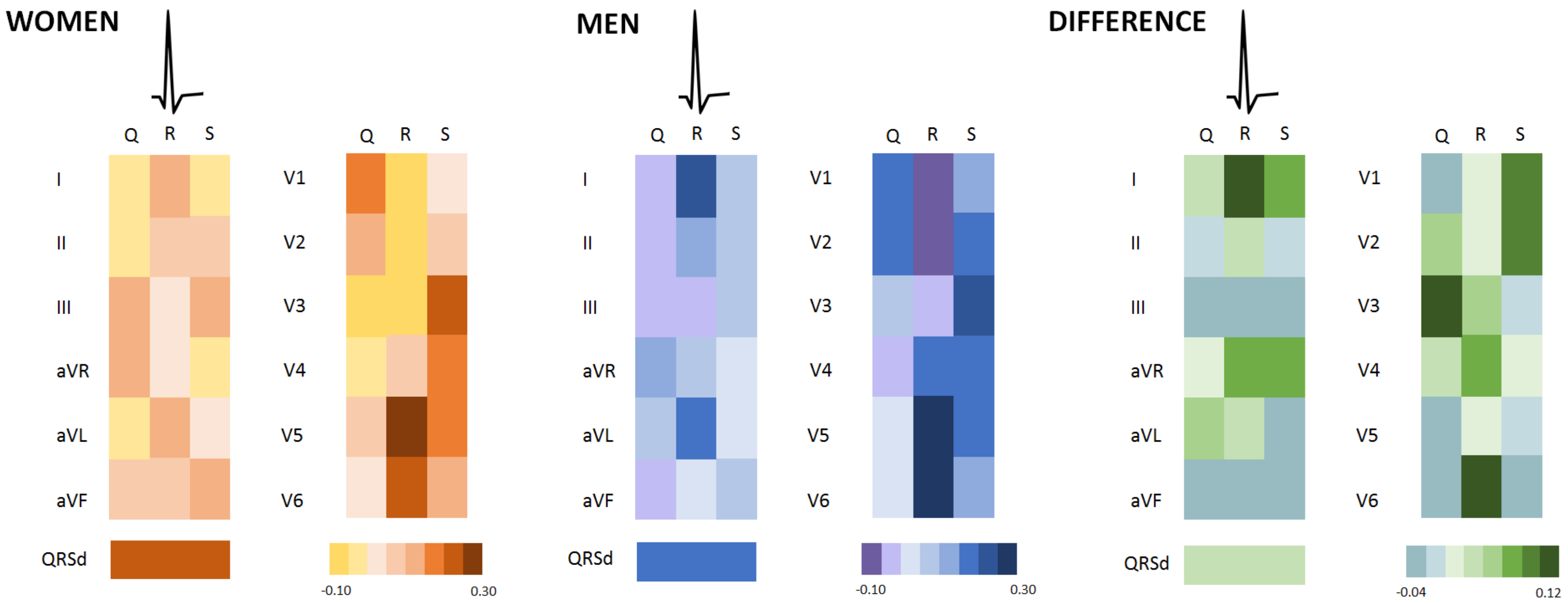

Online Figure 3. ROC curves for the existing and new developed ECG-LVH criteria in the **validation** cohort in women (left) and men (right).

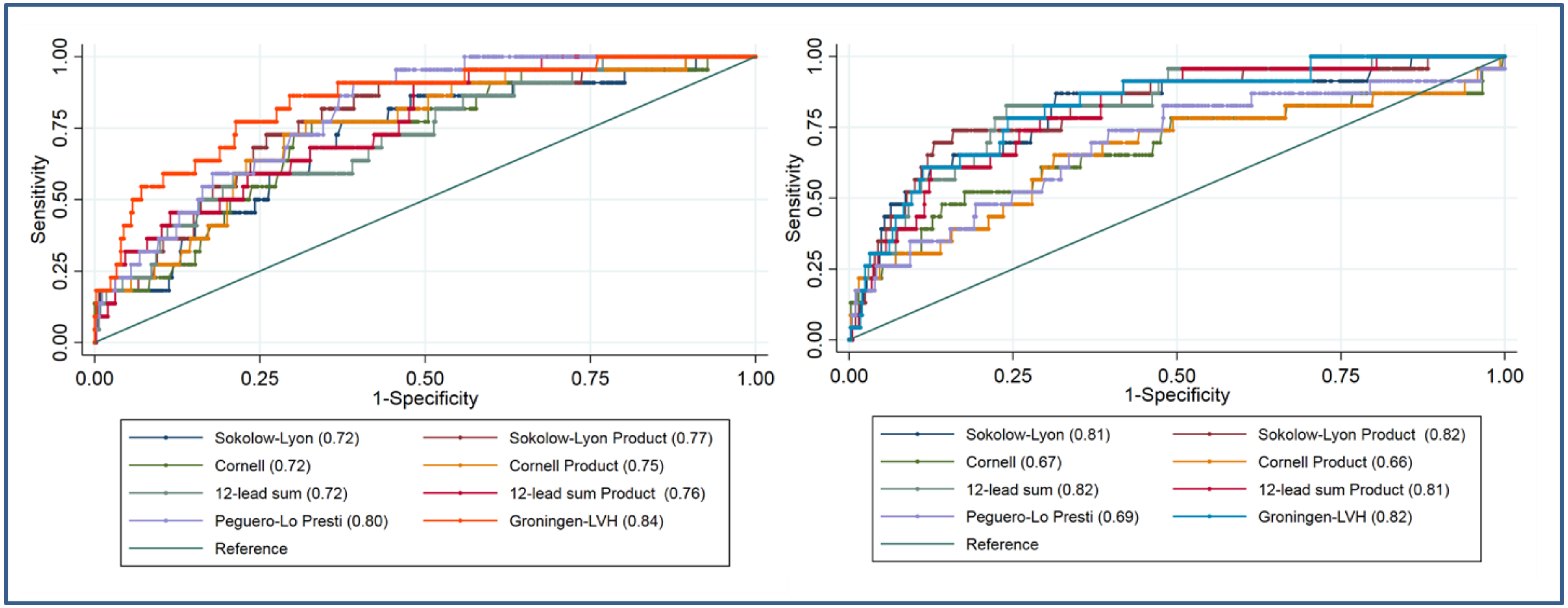

Supplement: Supplementary file 1 — Supplementary Information 1. [file 41598_2021_83137_MOESM1_ESM.pdf]
